# Food Retail Inequities in the Capital with Greatest Income Inequality in Brazil

**DOI:** 10.3390/nu18213472

**Published:** 2026-10-22

**Authors:** Joerika Ciqueira, Denise Sales, Patrícia Freitas, Cecília Craveiro, Aline Lopes, Mariana Lopes

**Affiliations:** 1 Observatório das Desigualdades em Alimentação e Nutrição, Departamento de Nutrição, Centro de Ciências da Saúde, Universidade Federal da Paraíba, Campus I, Cidade Universitária, João Pessoa 58051-900, PB, Brazil; joerikabatista@hotmail.com (J.C.); ceciliafc23@gmail.com (C.C.); 2 Fundação Oswaldo Cruz—Centro de Integração de Dados e Conhecimentos para Saúde/CIDACS, Parque Tecnológico da Bahia, R. Mundo, 121, Trobogy, Salvador 41745-715, BA, Brazil; denise.marques.sales@gmail.com; 3 Grupo de Pesquisa de Intervenções em Nutrição, Universidade Federal dos Vales do Jequitinhonha e Mucuri, Nutrition Building JK Campus—Highway BR 367—Km 583, Alto da Jacuba District, Diamantina 39100-000, MG, Brazil; patpfreitas@gmail.com; 4 Grupo de Pesquisa de Intervenções em Nutrição, Departamento de Nutrição, Universidade Federal de Minas Gerais, Escola de Enfermagem, 190 Alfredo Balena Avenue, Room 316, Santa Efigênia, Belo Horizonte 30130-100, MG, Brazil; alinelopesenf@gmail.com

**Keywords:** food environment, health inequities, spatial analysis, socioeconomic factors

## Abstract

**Background/Objectives**: The uneven distribution of food stores is a key determinant of health inequities. This ecological study aimed to map socioeconomic inequalities in the food environment of Joao Pessoa, Brazil. **Methods**: Stores were classified into three categories, according to the main type of food sold: unprocessed, processed, or ultra-processed. The Socioeconomic Index of Geographic Context for Health Studies (GeoSES) and its dimensions (Education, Income, Poverty, Material Deprivation, and Mobility) were used to analyze the territory. The association between the number of food stores and socioeconomic indicators was analyzed through thematic maps, correlation coefficients, and negative binomial regression models. **Results**: A total of 5087 food stores were mapped. A strong positive correlation was observed between the number of stores selling unprocessed foods and the GeoSES index (r = 0.85; *p* < 0.001), Education (r = 0.83; *p* < 0.001), Income (r = 0.85; *p* < 0.001), and Material Deprivation (r = 0.85; *p* < 0.001), while Mobility showed a moderate positive correlation (r = 0.59; *p* = 0.04). In the negative binomial regression model, each one-unit increase in the GeoSES index was associated with a 2.05-fold increase in the expected number of stores selling unprocessed foods (RR = 2.05; 95% CI: 1.48–2.84; *p* < 0.001). **Conclusions**: The food environment in Joao Pessoa, Brazil was associated with socioeconomic characteristics, with greater availability of unprocessed food stores in higher socioeconomic areas, while ultra-processed food stores were more evenly distributed across the municipality.

## 1. Introduction

The food environment is one of the key determinants of food choices and consumption patterns, with direct implications for population health and the sustainability of the food system [1,2]. At the community dimension, it encompasses the availability, type, location and accessibility of food retail establishments, as well as other factors that may shape food acquisition and consumption, including food affordability, marketing, consumer behavior, and the nutritional quality of available products [3].

Although the physical availability of food retail establishments is an important component of the community food environment, the presence of food retailers does not necessarily ensure access to healthy foods. Economic and financial accessibility may represent additional barriers, particularly in socioeconomically vulnerable areas, where healthy foods may be available but remain unaffordable for part of the local population. Therefore, physical availability and economic accessibility should be considered complementary dimensions of food access [3].

Unhealthy food environments, characterized by a greater availability of establishments offering predominantly unhealthy food options and a limited availability of healthier foods, may contribute to unhealthy dietary patterns and nutrition-related diseases. Dietary patterns characterized by a high consumption of ultra-processed foods have been associated with obesity and other noncommunicable diseases, including diabetes and cardiovascular diseases [4,5].

The distribution of food stores may not be random and it may reflect socioeconomic inequalities [6,7]. Unequal food environments are characterized by structural differences in food access, variety, quality and price of adequate and healthy food, thereby limiting the realization of the Human Right to Adequate Food (HRAF) [8,9]. In the United States of America, where 87.1% of all food environment studies were conducted, individuals belonging to racial and ethnical minorities are 70% more likely to live in areas considered food deserts [10].

International evidence further indicates that socioeconomic inequalities in food retail availability may vary substantially across urban contexts. Evidence from Latin America also suggests that these relationships may differ according to local socioeconomic and urban contexts. In Mexico, an analysis of changes in the retail food environment between 2010 and 2020 found that the growth of several types of food outlets, including supermarkets and fruit and vegetables stores, was greater in more socioeconomically deprived municipalities than in less deprived areas [11]. These findings suggest that socioeconomic inequalities in food retail environments do not necessarily follow a uniform pattern and may be shaped by local urban and commercial dynamics.

In Australia, a recent spatial analysis of metropolitan Melbourne and regional Victoria identified non-linear socioeconomic disparities in food outlet availability, with both highly disadvantaged and affluent areas showing higher densities of food outlets, while intermediate socioeconomic areas had lower availability. These findings highlight that the relationship between socioeconomic conditions and food retail distribution is context-dependent and may differ according to geographic setting and the type of food outlet considered [12].

Theses inequalities are also present in Brazilian cities. In Belo Horizonte, a major capital city, fruits and vegetables specialized stores—including public food and nutrition security facilities—are concentrated in wealthiest neighborhoods, while poorer areas faced a shortage of healthy food outlets [13]. However, establishments focused on the sale of ultra-processed foods (UPF) were present throughout the city, accounting for 46.9% of all food stores [13]. Similarly, in Jundiai, Sao Paulo, healthy food establishments were mainly located in central and wealthier areas [14].

In the Northeast of Brazil, at Recife, census tracts with few healthy food stores accounted for 28.5% of the urban area, concentrated in low income and education areas, and a higher proportion of Black and Indigenous populations. In contrast, census tracts with a predominance of establishments selling UPF accounted for 73.5% of the total and were predominantly located in more socioeconomically advantaged neighborhoods [15].

Although Brazilian ecological studies have advanced our understanding of the food environment, existing evidence has largely focused on major urban centers, particularly in the Southeast and South regions [6,13,14], although recent studies have also examined Northeastern capitals [15,16]. Moreover, previous investigations have frequently relied on isolated socioeconomic indicators, such as income or education, rather than multidimensional measures of socioeconomic context [6,14,15,16].

Although composite socioeconomic measures may provide a broader characterization of contextual inequalities, their application to food retail environments in Brazilian cities remains limited. This study expands the current literature by providing evidence from a Northeastern Brazilian capital, utilizing a comprehensive composite Socioeconomic Index of the Geographic Context for Health Studies (GeoSES) to assess socioeconomic inequalities in the spatial distribution of different food retail types across urban areas with contrasting socioeconomic contexts [17].

Therefore, Joao Pessoa constitutes a relevant locus for investigation, as it exhibits marked economic segregation [18], associated with rapid urban growth and characterized by profound territorial inequalities, making it the Brazilian state capital with the highest income inequality in the country [16,19]. These contrasts across the urban space make the city an informative setting for examining whether socioeconomic inequalities are reflected in the spatial distribution of different types of food retail establishments. Given this, this study aimed to map socioeconomic inequalities in the community food environment of the Joao Pessoa, by examining the spatial distribution of different types of food retail establishments and their association with the socioeconomic characteristics of the areas.

## 2. Materials and Methods

### 2.1. Study Design and Setting

This is an ecological study utilizing secondary data from food retail establishments from 2023–2024, in Joao Pessoa, Paraiba, Brazil, with GeoSES aggregation areas as the territorial units of analysis.

Joao Pessoa is the seventh most populous city in the Northeast region, and the twentieth in Brazil, with a population of 833,932, a land area of 210.04 km^2^, and a population density of 3970.27 people per km^2^ [20]. Although it has a Municipal Human Development Index (IDH-M) classified as high (0.763), the city is marked by socioeconomic inequalities [18]. According to data from the Brazilian Institute of Geography and Statistics [18], its Gini Index is 0.629, making it the capital city with the highest income inequality in Brazil. The Gini Index is a measure of income concentration, with values ranging from 0 (perfect equality) to 1 (maximum inequality) [21]. Beyond its high level of income inequality, Joao Pessoa is characterized by marked socioeconomic and territorial contrasts across its urban space [16,18,19], providing an informative setting to examine whether these contextual inequalities are reflected in the spatial distribution of different types of food retail establishments.

### 2.2. Data Collection

The investigation covered formal, public, and private food retail establishments in the city of Joao Pessoa. The list of private establishments was provided by the Paraiba State Department of Finance (SEFAZ-PB) and included information such as business name, address, corporate name, National Legal Entity Registration Number (CNPJ), and National Classification of Economic Activities (CNAE). Data on establishments involving public concessions and bids, such as district markets (with the exception of the Public Fish Market, provided by SEFAZ-PB), were supplied by the Joao Pessoa City Hall through the Secretariat of Urban Development and Control (Sedurb) via the “Prefeitura Conectada” electronic platform.

To ensure data quality and validate physical existence, a comprehensive virtual audit was conducted. All registered addresses were checked using Google Street View and updated satellite imagery in Google Earth Pro. The virtual verification was conducted by a trained research team following standardized procedures to verify the existence, location, and primary commercial activity of each establishment. Establishments identified as permanently closed or whose primary activity did not correspond to food sales were excluded from the database.

Official municipal records did not include information on open-air markets and kiosks, and had gaps in the address data, making it necessary to obtain this information through active research. For open-air markets, a search was conducted on municipal government websites and local news portals. For kiosks, data collection was carried out using a mixed-method approach, combining virtual audits of public spaces, squares, and the waterfront using satellite imagery and Google Street View with in-person verification of beachfront kiosks to confirm their physical presence and commercial activity during the study period.

The following exclusion criteria were used to compile the database: establishments for which food sales were not the primary business activity, such as hotels, pharmacies, and department stores; catering services, due to their predominantly on-demand nature and focus on specific events; and street vendors, due to the lack of a fixed sales location and high spatial turnover, which would make proper verification impractical. Specifically for this study, community restaurants and kitchens, solidarity kitchens, and food banks were ineligible because they operate under a social protection model, with subsidized prices or free distribution, differing structurally from the dynamics of free-market sales, which is the focus of this study.

All establishments were mapped in Google Earth Pro version 7.3.7 (Mountain View, CA, USA) using the WGS 84 geographic coordinate system and georeferenced in QGis 3.34 (Anchorage, AK, USA).

The classification of food establishments was based on the Locais-Nova system for the Northeast of Brazil, which categorizes establishments into: (1) unprocessed foods; (2) processed foods; and (3) UPF. Establishments offering a variety of food products were classified according to their commercial activity and the predominant food profile associated with each category in the Locais-Nova system, rather than based on a complete inventory of foods available at each individual establishment. Thus, the classification reflected the predominant food profile associated with each type of commercial activity. Since seaside kiosks are not cataloged as a distinct category in official records, an operational adjustment was made: kiosks that sell exclusively coconut water were classified as establishments selling predominant fresh foods, while general kiosks selling drinks and snacks were classified as establishments selling predominantly UPF, reflecting their main commercial offer (Table S1 in the Supplementary Materials) [22].

### 2.3. Socioeconomic Index of the Geographic Context for Health Studies (GeoSES)

GeoSES was used to conduct a socioeconomic characterization of the territory, with five of its seven dimensions. GeoSES is a validated composite indicator based on data from the 2010 Demographic Census that integrates multiple census variables, providing a robust and multidimensional representation of the socioeconomic context. The index comprises the following dimensions: Education, Mobility, Poverty, Income, and Material Deprivation. In this study, the Wealth and Segregation dimensions were not analyzed separately. This decision was made specifically for the present analysis to avoid redundancy between the Wealth and Poverty dimensions. Furthermore, the Segregation dimension involves high conceptual complexity, requiring specific research and discussions that go beyond the scope of this study [17]. Although the exclusion of these dimensions may limit the assessment of specific aspects of socioeconomic inequalities, the five dimensions included in the analysis allowed for a multidimensional characterization of the socioeconomic context [17].

GeoSES scores range from −1 (worst conditions) to +1 (best conditions), with a score of 0 representing the average for the geographic area under analysis. Thus, positive values indicate that the area is above average, and negative values indicate that it is below average [17]. In its general methodological framework for the dimensions of Education, Income, and Mobility, higher values represent better socioeconomic conditions, whereas for the dimensions of Poverty and Material Deprivation, the analysis is reversed, with higher values indicating worse socioeconomic conditions [17].

In this study, GeoSES aggregation areas were used as the territorial unit, as they ensure greater statistical stability and temporal comparability compared to individual census tracts [17].

### 2.4. Data Analysis

The association between the number of food retail establishments and the GeoSES index and its five socioeconomic dimensions was analyzed using thematic maps, Pearson’s correlation coefficient, and negative binomial regression models (*p*-value < 0.05), using Stata 13.0 (College Station, TX, USA) and ArcGIS 10.5 (Redlands, CA, USA). Separate negative binomial regression models were fitted for the overall GeoSES index and for each of its five socioeconomic dimensions. Thus, each socioeconomic indicator was analyzed independently, rather than being included simultaneously with the other dimensions in the same model.

The establishments, classified according to the type of food predominantly sold, were overlaid onto GeoSES aggregation areas and stratified into quintiles. The 12 GeoSES aggregation areas were distributed across the five socioeconomic quintiles as follows: two areas in the lowest quintile, two in the second quintile, four in the intermediate quintile, two in the fourth quintile, and two in the highest quintile. The use of the aggregation area rather than census tracts was based on the fact that the latter scale would result in a reduced count of establishments per territorial unit, which would compromise the robustness of the spatial and statistical analyses [17].

The number of establishments per aggregation area was represented at the centroid of each area using circles proportional to the number of facilities. These circles were scaled to ensure equal intervals between the categories of facilities represented.

The initial association was assessed using Pearson’s correlation coefficient. Next, Negative Binomial Regression with a log-link function was applied due to overdispersion in the count data (variance exceeding the conditional mean; likelihood-ratio test *p* < 0.001). To account for variations in population size across aggregation areas, the natural logarithm of the total resident population in each area was included as an exposure offset term in all models. Consequently, the models estimated population-adjusted Rate Ratios (RRs) and their respective 95% Confidence Intervals (95% CI) and McFadden’s Pseudo R^2^. Statistical significance was set at *p* < 0.05.

### 2.5. Ethical Aspects

This study used only secondary data in the public domain that were not individually identifiable; therefore, it was exempt from approval by a Research Ethics Committee, in accordance with Resolution No. 466/12 of the National Health Council.

## 3. Results

The municipality of Joao Pessoa consists of 12 aggregation areas, and a total of 5087 food retail establishments were included in the final analysis. Areas with higher education and income indicators are concentrated in the eastern part of the city, encompassing neighborhoods located near the waterfront. In contrast, the northwest and far south regions have the lowest education indicators, followed by the west region. In these regions, the lowest income levels and highest levels of poverty and material deprivation were also observed.

The majority were classified as predominantly selling UPF (69.9%; *n* = 3554), followed by those predominantly selling unprocessed foods (24.7%; *n* = 1259) and processed foods (5.4%; *n* = 274).

**Figure 1:**
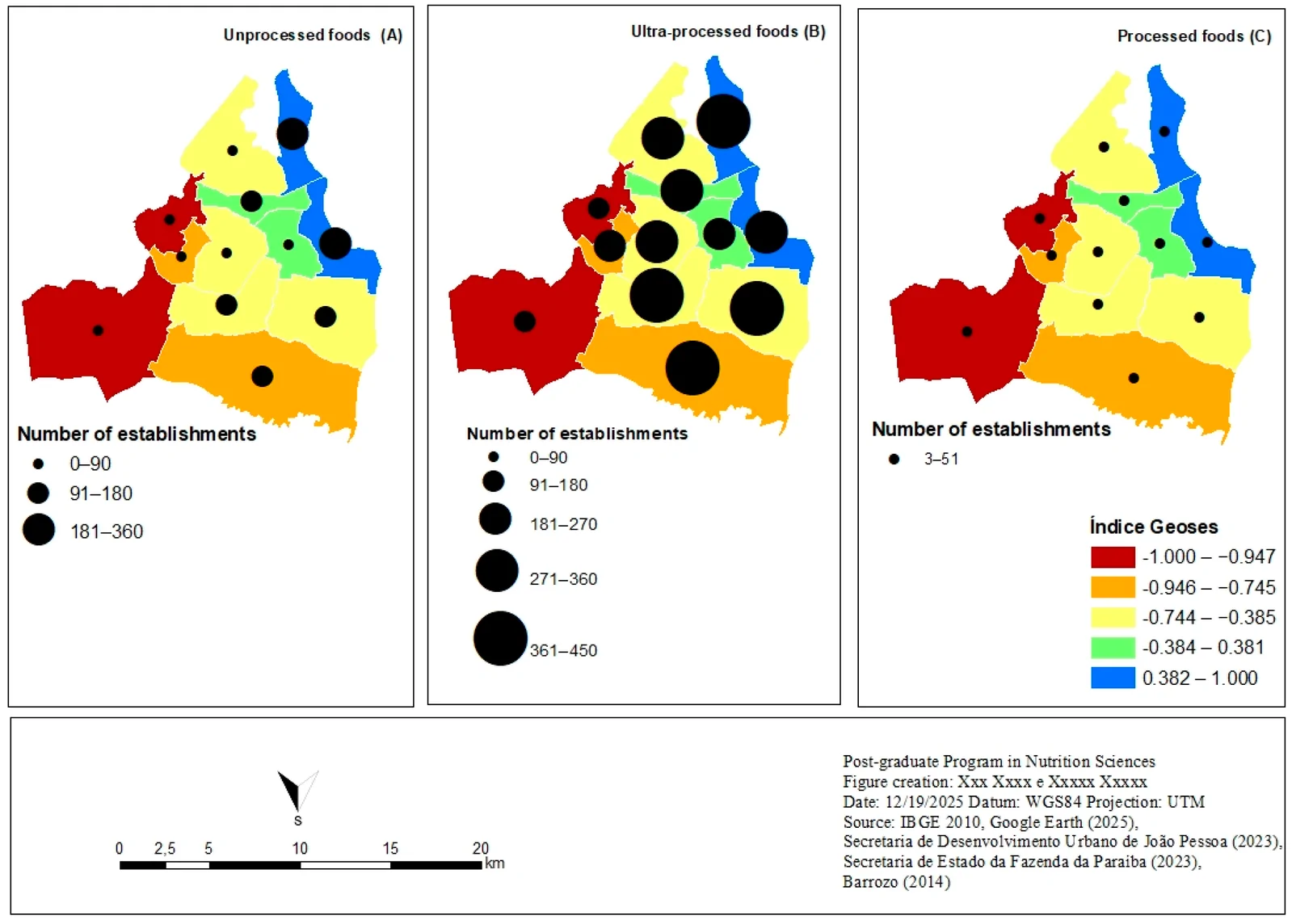
Spatial distribution of types of food retails establishments according to the GeoSES index, Joao Pessoa, Brazil, 2023–2024. (**A**) Unprocessed foods; (**B**) Ultra-processed foods; (**C**) Processed foods. Sources: IBGE [20]; Google Earth; Joao Pessoa Secretariat of Urban Development and Control; Joao Pessoa State Department of Finance; Barrozo et al. [17].

The spatial distribution of establishment types according to the GeoSES index is shown in Figure 1A–C. A lower absolute number of establishments selling unprocessed foods—represented by smaller circles (0–90)—was observed in the clusters with the lowest GeoSES index, which geographically correspond to the city’s outlying areas. On the other hand, the highest concentrations of this type of establishment were identified in the coastal and central regions of the city, areas with higher Income brackets (up to R$ 9138.40) and Education levels (up to 35,492) (Figure 1A).

The density of establishments primarily selling UPF was high throughout the city, with larger circles (361–450 establishments) located in densely populated areas with intermediate to high GeoSES values, situated in the city’s coastal and central geographic regions. In areas with lower GeoSES values, the physical presence of UPF stores was higher (91–180) compared to stores selling unprocessed foods (Figure 1A). In contrast, food outlets selling processed foods (Figure 1C) presented lower physical density across all areas and appeared to be distributed more evenly across the city. This widespread distribution of establishments selling UPF contrasted with the unequal spatial distribution of establishments selling unprocessed foods, which were less available in areas with lower socioeconomic status.

**Figure 2:**
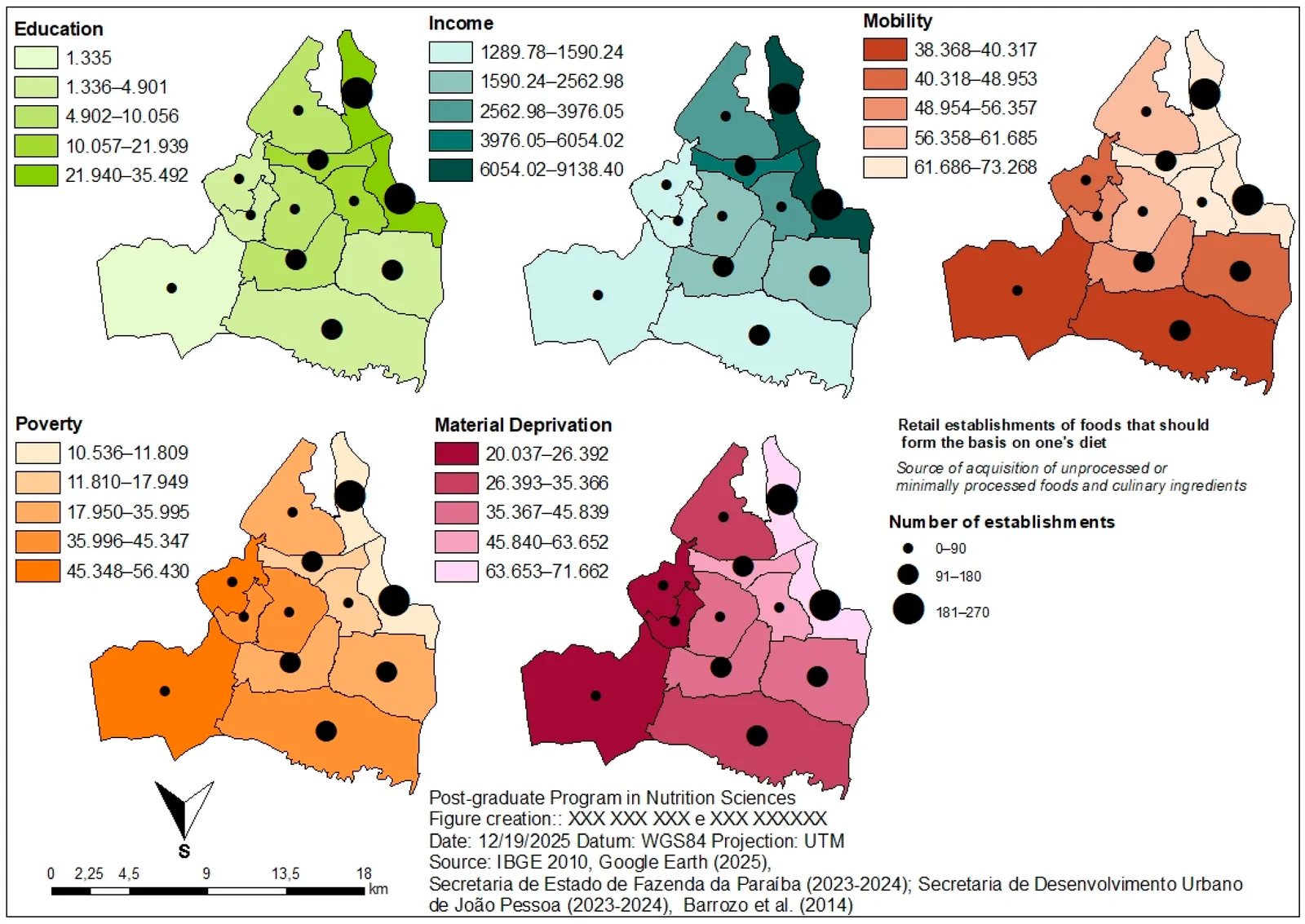
Distribution of food retail establishments selling primarily unprocessed foods according to the GeoSES index dimensions, Joao Pessoa, Brazil, 2023–2024. Sources: IBGE [20]; Google Earth; Joao Pessoa Secretariat of Urban Development and Control; Joao Pessoa State Department of Finance; Barrozo et al. [17].

Figure 2 shows the spatial distribution of establishments selling unprocessed foods according to the dimensions assessed. Most of the establishments, represented by the larger circles, were in the city’s coastal and central geographic regions. Higher levels of Poverty (up to 56,430) and Material Deprivation (up to 71,662) were observed mainly in the western, northwestern, and central-western regions of the city.

**Figure 3:**
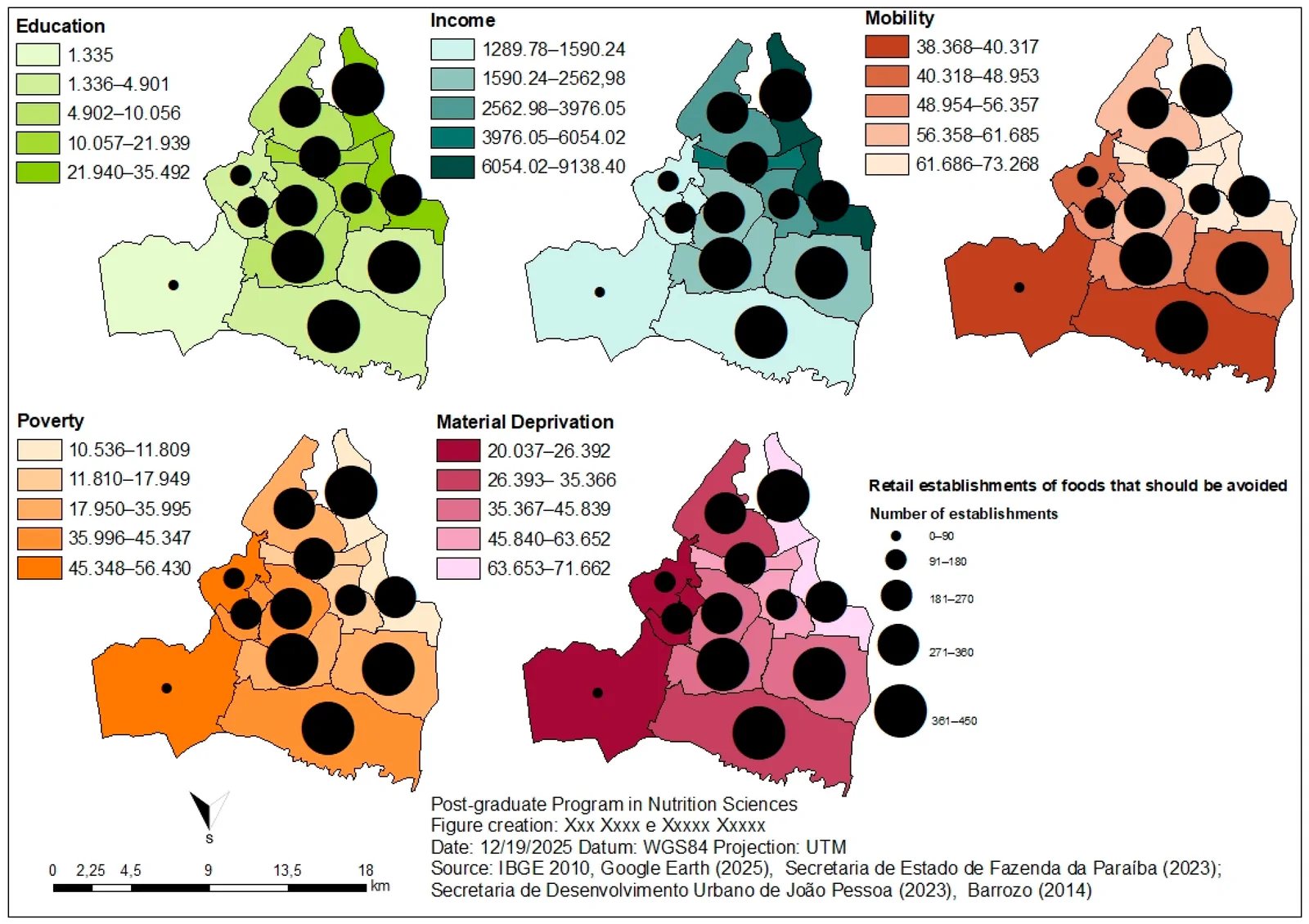
Distribution of food retail establishments selling primarily ultra-processed foods according to the GeoSES index dimensions, Joao Pessoa, Brazil, 2023–2024. Sources: IBGE [20]; Google Earth; Joao Pessoa Secretariat of Urban Development and Control; Joao Pessoa State Department of Finance; Barrozo et al. [17].

**Figure 4:**
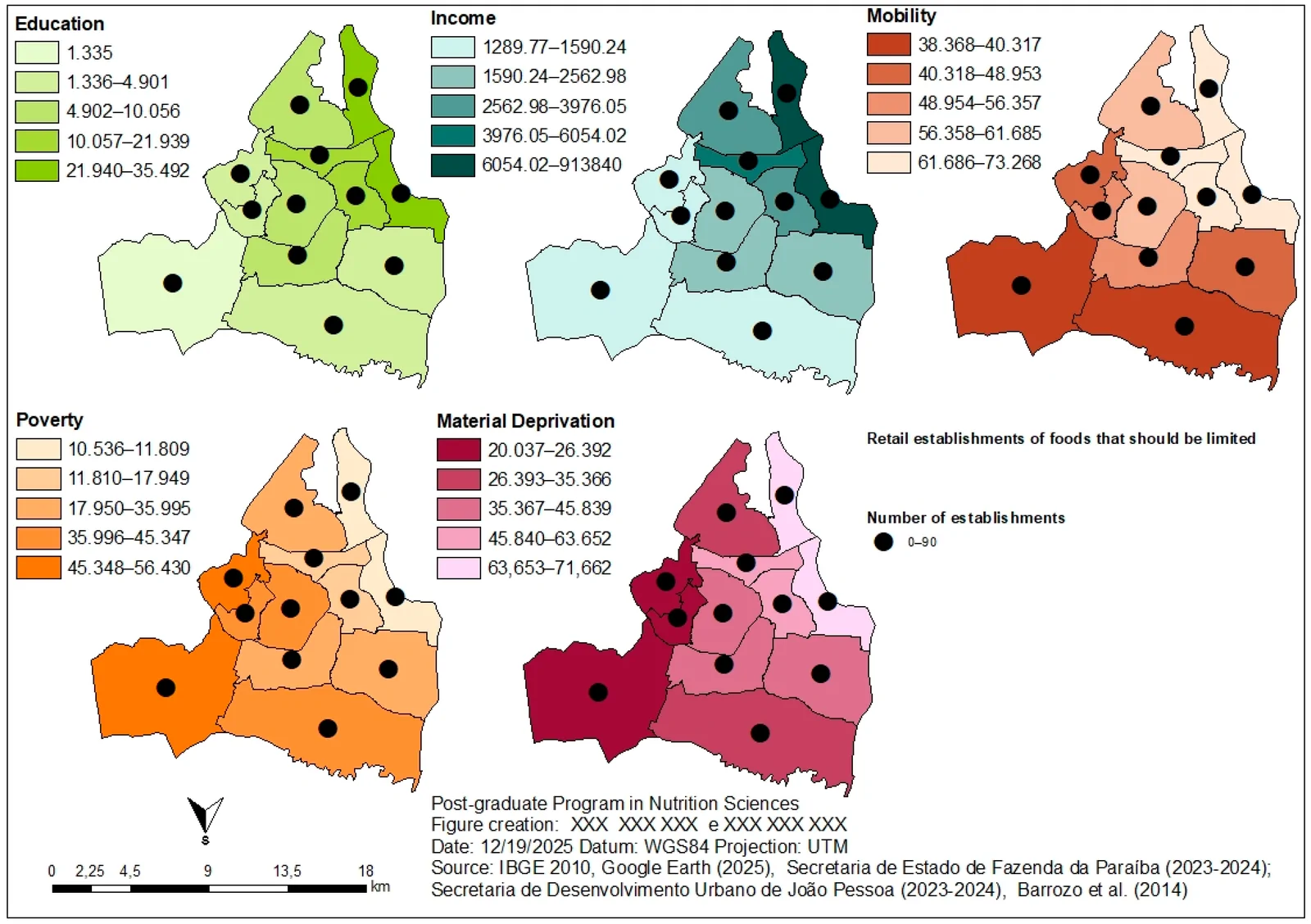
Distribution of food retail establishments selling primarily processed foods according to the GeoSES index dimensions, Joao Pessoa, Brazil, 2023–2024. Sources: IBGE [20]; Google Earth; Joao Pessoa Secretariat of Urban Development and Control; Joao Pessoa State Department of Finance; Barrozo et al. [17].

UPF outlets were concentrated throughout the city, with few differences across the GeoSES areas. However, a lower spatial concentration of outlets (ranging from 0–90) was observed in a specific area located at the western edge of the city, a pattern that remained consistent across all vulnerability scenarios evaluated (Figure 3). Food outlets selling processed food were uniformly distributed across the city, falling in the lower range of the scale (0–90 stores) (Figure 4).

The correlation analyses were based on the number of food retail establishments within each GeoSES aggregation area. A strong positive correlation was observed between the availability of establishments predominantly selling unprocessed foods and the overall GeoSES index (r = 0.85; *p*-value < 0.001), Education (r = 0.83; *p*-value < 0.001), Income (r = 0.85; *p*-value < 0.001), and Material Deprivation (r = 0.85; *p*-value < 0.001). Mobility was also positively correlated, although to a moderate extent (r = 0.59; *p*-value = 0.04), (Table S2 in the Supplementary Materials).

Regarding model performance, McFadden’s pseudo-R^2^ values ranged from 0.00 to 0.11 across the negative binomial regression models. Unlike the coefficient of determination (R^2^) in ordinary least squares regressions, pseudo-R^2^ measures proportional improvement in model log-likelihood and typically yields substantially lower values in ecological count regressions. In urban food environments, commercial location choices are driven by complex unmeasured spatial determinants beyond area-level socioeconomic status—such as municipal commercial zoning regulations, commercial rental land values, urban road hierarchy, and pedestrian transit corridors—which inherently leaves substantial spatial variance uncaptured by census indicators alone.

In the Negative Binomial Regression models, each one-unit increase in the GeoSES index was associated with a 2.05-fold increase in the expected number of establishments selling unprocessed foods (RR = 2.05; 95% CI: 1.48–2.84; *p*-value < 0.001). Associations with the individual dimensions of the GeoSES index were interpreted according to their respective scales of measurement and were not directly compared with the composite index (Table 1). This association reflects differences in the physical availability of food retail establishments across areas and should not be interpreted as evidence of greater overall food access, as economic accessibility, transportation, and other dimensions of food access were not assessed in this study.

## 4. Discussion

The community food environment in Joao Pessoa exhibits pronounced territorial disparities in the physical availability of food retailers, with areas of higher socioeconomic status, particularly the city’s central and coastal areas, having more than twice the density of retail establishments selling unprocessed foods. On the other hand, commercial establishments selling processed foods and UPF were distributed throughout the city, regardless of the area’s socioeconomic status.

The wide spatial availability and density of processed foods and UPF observed throughout the city may represent an obesogenic feature of the food environment, given the widespread presence of these food products across the territory [4]. In this context, the unequal physical availability of establishments selling unprocessed foods may contribute to socioeconomic inequalities in the food environment, particularly in vulnerable areas where healthier retail options are less available. This may be even more impactful in the most vulnerable areas which lack access to healthy foods. The rise in UPF consumption has been identified as one of the main determinants of the increasing prevalence of obesity and other chronic noncommunicable diseases worldwide [5].

The spatial concentration of all types of food retail establishments in areas with better socioeconomic indicators suggests a community food environment with higher commercial density and a wider diversity of retail food options. A similar pattern was observed in another capital city in Northeastern Brazil, Fortaleza, where the highest concentration of commercial establishments was found in areas with higher IDH-M and average household income [16].

Similar socioeconomic disparities have also been documented in international settings, although the relationship between socioeconomic conditions and food retail availability may not always follow a linear gradient. In metropolitan Melbourne and regional Victoria, Australia, Marzan et al. found that both highly disadvantaged and affluent areas had higher densities of food outlets, whereas areas with intermediate socioeconomic conditions consistently showed lower availability. The study also identified geographic differences, with disadvantaged rural areas experiencing limited availability of healthy food outlets and regional urban centers showing high densities of less healthy options. These findings reinforce that socioeconomic inequalities in food environments are context-dependent and shaped by the interaction between social disadvantage, urban structure, and geographic location [12].

These results suggest that inequalities in the food environment are not an isolated regional phenomenon, but rather a structural feature of Brazil’s unequal urbanization process. However, the extent of these inequalities may vary across the country’s macro-regions. The Summary Brazilian National Mapping of Food Deserts and Food Swamps [23] highlighted this regional heterogeneity, with greater territorial inequalities in the North and Northeast regions. These two macro-regions encompassed approximately 86% of Brazilian municipalities with the lowest densities of healthy food retail establishments, possibly indicating an even greater scarcity of healthy food supply in the poorest areas of these municipalities.

Although the rapid growth of Brazilian cities is characterized by specific patterns of spatial segregation, the unequal distribution of food retailers reflects a broader market-driven logic observed across diverse urbanizing contexts globally.

An analysis of the specific dimensions comprising the GeoSES index can provide further insight into how socioeconomic characteristics are associated with the distribution of food retail establishments across the territory. Unlike isolated socioeconomic indicators, the multidimensional structure of the GeoSES index allows socioeconomic context to be assessed across multiple interconnected dimensions, capturing territorial inequalities that may not be fully represented by income, education, or other single indicators alone. The positive correlations identified between the GeoSES index and the dimensions of Education, Income, Material Deprivation, and Mobility and the presence of retail establishments selling unprocessed foods indicate that the distribution of these establishments varied according to the socioeconomic characteristics of the areas. For Material Deprivation, this positive correlation indicates that areas with higher levels of material deprivation also had a higher number of establishments selling unprocessed foods, consistent with the direction of this GeoSES dimension.

At the contextual level, higher neighborhood income reflects greater aggregate purchasing power and may be associated with more favorable conditions for commercial investments in diverse and perishable fresh food retail. While individual purchasing behavior was not directly measured, areas with more favorable socioeconomic characteristics, particularly higher income and education, showed greater physical availability of food retail establishments [24]. On the other hand, socially vulnerable territories were characterized by lower economic indicators and lower availability of establishments selling healthy foods, indicating important territorial inequalities in the distribution of these establishments [24].

In addition, poor transport infrastructure may further constrain mobility and access to healthy food. As noted by Rocha et al. [25], reaching open-air markets and grocery stores in vulnerable areas may require longer travel times when using public transportation. In Joao Pessoa, limitations in public transportation may interact with spatial patterns of food retail availability. In this context, areas characterized by lower mobility and lower availability of establishments selling unprocessed foods may present greater territorial constraints on the distribution of healthy food retail [25].

Duarte et al. [26], in their analysis of the city of Sao Paulo, found that the lack of basic urban infrastructure posed logistical challenges that also hindered formal commerce. Among the obstacles identified, inadequate paving stood out, as it increases costs and hinders the transport of goods, as well as instability in the electrical grid—elements that constitute the Material Deprivation construct of the GeoSES Index and may represent infrastructural constraints for the distribution and preservation of fresh foods in vulnerable areas. Thus, in Joao Pessoa, infrastructural conditions may interact with territorial patterns of food retail availability, although the specific relationship may vary across socioeconomic dimensions.

Despite its methodological strengths, this study has limitations that must be considered. The commercial data were largely derived from the SEFAZ-PB administrative registry, which is subject to time lags inherent to tax records relative to real-time retail dynamics, as business closures or changes in primary commercial activities are not always promptly reported. However, this administrative limitation was substantially mitigated by conducting a comprehensive virtual audit of all registered addresses using Google Street View and updated satellite imagery in Google Earth Pro (2023–2024), effectively filtering out permanently closed or misclassified establishments and ensuring high data accuracy.

Another limitation involves the temporal mismatch between the socioeconomic indicators used in the GeoSES index (derived from the 2010 Demographic Census) and the food retail database (audited in 2023–2024), as detailed intra-urban socioeconomic microdata from the 2022 Census were not yet released at the time of analysis. The extensive intercensal window was characterized by profound macroeconomic shifts, including the socioeconomic fallout of the coronavirus disease 2019 (COVID-19) pandemic, which disproportionately affected income and employment among vulnerable populations [27]. Therefore, urban expansion and socioeconomic transformations that occurred between 2010 and 2024 may have modified the socioeconomic characteristics of some areas, and the GeoSES classification may not fully represent the socioeconomic context of Joao Pessoa at the time of food retail data collection. Future investigations should update these spatial metrics as new census tract-level socioeconomic data become available to capture subsequent urban developments.

The small number of geographic units analyzed, comprising only 12 aggregation areas, also represents an important limitation. This limited number of observations may reduce the precision of the estimated associations and affect statistical inference and should therefore be considered when interpreting the regression results. In addition, spatial autocorrelation was not formally assessed, and therefore potential spatial dependence among neighboring areas cannot be ruled out. This may affect the independence assumption underlying conventional regression inference and the precision of the estimated associations.

In addition, the ecological design of this study does not allow individual-level inferences, and the observed associations should not be interpreted as causal relationships. The study focused on the physical availability and spatial distribution of food retail establishments and did not assess food prices, food quality, affordability, or actual food purchasing and consumption behaviors. Therefore, the presence of an establishment in a given area does not necessarily indicate that its products are economically accessible, of adequate quality, or effectively purchased by the local population. Future studies incorporating individual-level data and information on food prices, quality, and purchasing behaviors could provide a more comprehensive understanding of food access and its relationship with socioeconomic inequalities.

Thus, this study stands out for its pioneering approach to mapping the community food environment in Joao Pessoa and for using the aggregation areas of the GeoSES index as a unit of analysis, providing important insights into local inequities [17]. Furthermore, the use of the Locais-Nova system to classify food retail establishments was noteworthy because it was based on the guidelines of the Dietary Guidelines for the Brazilian Population [28].

The identification of areas with low availability of healthy food retail establishments and a high concentration of establishments predominantly selling UPF highlights territorial conditions that may be relevant to discussions on the human right to adequate food (HRAF) and food and nutrition security.

Therefore, municipal urban planning must structurally integrate food supply with food and nutrition security policies, prioritizing the establishment of public food facilities (such as public markets, popular restaurants, and community gardens) alongside built environment and infrastructure improvements in vulnerable areas. Although Joao Pessoa has an established network of food and nutrition security initiatives [29], strategic planning remains essential to ensure the equitable territorial distribution of these facilities across the urban space.

## 5. Conclusions

The municipality’s food environment was associated with the socioeconomic characteristics of its territory, with greater availability of establishments selling unprocessed foods in areas with higher socioeconomic status. In contrast, establishments predominantly selling ultra-processed foods were more evenly distributed across the municipality. The findings may support the development of location-specific and multisectoral policies aimed at improving the physical availability of establishments selling unprocessed foods in underserved areas and integrating food environment considerations into urban planning and food and nutrition security policies in Joao Pessoa, Brazil.

## Figures and Tables

**Table 1:** Negative Binomial Regression Models for the association between the GeoSES index and its dimensions and types of food retail establishments. Joao Pessoa, Brazil. 2023–2024.

Types of Food Retail Establishments	Variables	RR	95% CI	*p*-Value	Pseudo R^2^
Unprocessed foods	GeoSES	2.05	1.48–2.84	<0.001	0.09
	Education	1.000036	1.000017–1.000055	<0.001	0.08
	Income	1.000000	1.000000–1.000000	<0.001	0.08
	Poverty	0.999774	0.999556–0.999992	0.042	0.03
	Material Deprivation	1.000029	1.000018–1.000040	<0.001	0.11
	Mobility	1.000310	1.000060–1.000550	0.013	0.04
Ultra-processed foods	GeoSES	1.25	0.85–1.85	0.264	0.01
	Education	1.000009	0.999984–1.000030	0.386	0.01
	Income	1.000000	0.999999–1.000000	0.35	0.01
	Poverty	0.999976	0.999779–1.000170	0.81	0.00
	Material Deprivation	1.000011	0.999964–1.000026	0.14	0.01
	Mobility	1.000040	0.999813–1.000028	0.71	0.00
Processed foods	GeoSES	0.95	0.49–1.82	0.866	0.00
	Education	0.999994	0.999602–1.000270	0.709	0.00
	Income	1.000000	0.999998–1.000000	0.785	0.00
	Poverty	1.000150	0.999843–1.000460	0.332	0.01
	Material Deprivation	1.000030	0.999771–1.000280	0.834	0.00
	Mobility	1.000030	0.999771–1.000280	0.371	0.01

Notes: All models included the natural logarithm of the resident population as an offset variable. RRs = Rate Ratios; 95% CI = 95% Confidence Interval. McFadden’s Pseudo-R^2^ evaluates model goodness-of-fit based on proportional improvement in log-likelihood relative to the null model, where lower values are typical in ecological spatial count regression models.

## Data Availability

The raw data supporting the conclusions of this article will be made available by the authors on request, due to authors’ privacy.

## Supplementary Materials

The following supporting information can be downloaded at: https://www.mdpi.com/article/10.3390/nu18213472/s1, Table S1: Types and descriptions of food retail stores in Joao Pessoa, Brazil, 2023–2024; Table S2: Pearson’s correlation between GeoSES Index and its dimensions with food retail stores types’ availability in Joao Pessoa, Brazil, 2023–2024.

## Abbreviations

The following abbreviations are used in this manuscript:

GeoSES	Socioeconomic Index of Geographic Context for Health Studies
HRAF	Human Right to Adequate Food
IDH-M	Municipal Human Development Index
UPF	Ultra-processed foods

## References

[1] HLPE High Level Panel of Experts on Food Security and Nutrition (2017). Nutrition and Food Systems: A Report by the High Level Panel of Experts on Food Security and Nutrition of the Committee on World Food Security.

[2] United Nations Transformando Nosso Mundo: A Agenda 2030 para o Desenvolvimento Sustentável. https://brasil.un.org/sites/default/files/2020-09/agenda2030-pt-br.pdf.

[3] Downs S.M., Ahmed S., Fanzo J., Herforth A. (2020). Food Environment Typology: Advancing an Expanded Definition, Framework, and Methodological Approach for Improved Characterization of Wild, Cultivated, and Built Food Environments toward Sustainable Diets. Foods.

[4] Louzada M.L.D.C., Costa C.D.S., Souza T.N., Cruz G.L.D., Levy R.B., Monteiro C.A. (2021). Impact of the consumption of ultra-processed foods on children, adolescents and adults’ health: Scope review. Cad. Saúde Pública.

[5] World Health Organization (2003). Diet, Nutrition, and the Prevention of Chronic Diseases: Report of a Joint WHO/FAO Expert Consultation.

[6] Duran A.C., Diez Roux A.V., Latorre M.D.R.D.O., Jaime P.C. (2013). Neighborhood Socioeconomic Characteristics and Differences in the Availability of Healthy Food Stores and Restaurants in Sao Paulo, Brazil. Health Place.

[7] Mackenbach J.D., Nelissen K.G.M., Dijkstra S.C., Poelman M.P., Daams J.G., Leijssen J.B., Nicolaou M. (2019). A Systematic Review on Socioeconomic Differences in the Association between the Food Environment and Dietary Behaviors. Nutrients.

[8] Ziso D., Chun O.K., Puglisi M.J. (2022). Increasing Access to Healthy Foods through Improving Food Environment: A Review of Mixed Methods Intervention Studies with Residents of Low-Income Communities. Nutrients.

[9] Craveiro C., Lopes M., Freitas P., Lopes A. (2025). Neighborhood Vulnerability and the Consumer Food Environment in an Urban Area. Int. J. Environ. Res. Public Health.

[10] da Silva Magalhães E.I., Schattschneider D.P., de Vargas E.B., da Silva L.Y., Borges D.C., da Conceição W.L., Fanton M., Canuto R. (2025). Ethnic-Racial Disparities in Food Environments: A Systematic Review and Meta-analyses of Inequities in Food Access and Availability. J. Racial Ethn. Health Disparities.

[11] Ramírez-Toscano Y., Pérez-Ferrer C., Bilal U., Auchincloss A.H., Barrientos-Gutierrez T. (2022). Socioeconomic deprivation and changes in the retail food environment of Mexico from 2010 to 2020. Health Place.

[12] Marzan M.B., Hui L., Jiang H., Takele W.W., de Leon M., Nacpil J.C., Wang Y., Wake M., Mavoa S. (2025). Non-Linear Socioeconomic Inequality in the Distribution of Food Outlets in Metropolitan Melbourne and Regional Victoria, Australia. Health Promot. J. Austr..

[13] Lopes A.C.S., Menezes M.C.D., Araújo M.L.D. (2017). O ambiente alimentar e o acesso a frutas e hortaliças: “Uma metrópole em perspectiva”. Saude Soc..

[14] Fortes M.F., Borges C.A., Miranda W.C., Jaime P.C. (2018). Mapeando as desigualdades socioeconômicas na distribuição do comércio varejista local. Segur. Aliment. Nutr..

[15] Lima da Silva J.M., Oliveira J.S., Casagrande Borges D., Honório O.S., Mendes L.L., Canuto R. (2024). Social inequities in food deserts and food swamps in a northeastern Brazilian capital. J. Biosoc. Sci..

[16] Barbosa B.B., Penha E.D.D.S., Carioca A.A.F. (2022). Food Environment of the Economic Capital of the Northeast: Social and Territorial Disparities in the Availability of Food Stores. Rev. Nutr..

[17] Barrozo L.V., Fornaciali M., De André C.D.S., Morais G.A.Z., Mansur G., Cabral-Miranda W., De Miranda M.J., Sato J.R., Amaro Júnior E. (2020). GeoSES: A Socioeconomic Index for Health and Social Research in Brazil. PLoS ONE.

[18] IBGE–Instituto Brasileiro de Geografia e Estatística (2024). Joao Pessoa (PB)—Cidades e Estados.

[19] Cruz M.P.M.D., Araújo T.J.M., Vasconcelos R.L.A., Mariano Ipolito A.L., Gomes De Asevedo M.D. (2024). Um Retrato da Segurança Alimentar do Nordeste Brasileiro no Período 2004 a 2018. Estudo Debate.

[20] IBGE–Instituto Brasileiro de Geografia e Estatística (2022). Brazil/Paraiba/Joao Pessoa: Panorama.

[21] Medeiros M. (2012). Medidas de Desigualdade e Pobreza.

[22] Silva M.A.L., Mendes L.L., Leite M.A., Rocha L.L., Borges C.A., Levy R.B., Louzada M.L.D.C. (2025). Food purchasing places classification system based on the Dietary Guidelines for the Brazilian Population: Locais-Nova. Epidemiol. Serv. Saude.

[23] Brasil Ministério do Desenvolvimento e Assistência Social, Família e Combate à Fome (2024). Sumário Mapeamento dos Desertos e Pântanos Alimentares: Principais Achados.

[24] Viecelli P.C., Gazolla M. (2021). Perspectivas contemporâneas do consumo de alimentos: Compreendendo grupos socioeconômicos de consumidores. Coloquio.

[25] Dantas N.F.B.F. (2023). Transporte Público por Ônibus e Segregação Urbana: Um Estudo Sobre Acesso a Oportunidades em João Pessoa-PB. Master’s Thesis.

[26] Duarte A.L.D.C.M., Rodrigues V.P., Alves R.C.F., Oliveira G.M.D. (2024). Access to fresh food in vulnerable urban areas: A classification study of the favelas and formal establishments in São Paulo. Rev. Adm. Pública.

[27] Ruptura, Retrocesso e Desigualdade: Como Entender o Período Intercensos 2010–2022?. https://www.nepp.unicamp.br/wp-content/uploads/sites/57/2025/03/CadPesq_97.pdf.

[28] Ministerio da Saude (2014). Dietary Guidelines for the Brazilian Population.

[29] Brasil Ministério do Desenvolvimento e Assistência Social, Família e Combate à Fome (2025). Cadernos de Experiências Sobre Políticas Alimentares Urbanas: Estratégia Alimenta Cidades.

