# Supplementary material for: Food Retail Inequities in the Capital with Greatest Income Inequality in Brazil
Source: Nutrients. 2026 Oct 22;18(21):3472. doi: 10.3390/nu18213472 (PMC13765393; doi:10.3390/nu18213472)
Supplement: Supplementary file 1 [file nutrients-18-03472-s001.zip › nutrients-4452111-supplementary.pdf]

Table S1. Types and descriptions of food retail stores in Joao Pessoa, Brazil, 2023–2024.

Locais-Nova Categories	Commercial Activities
Stores predominantly selling fresh foods	Retail sale of general items (supermarkets and hypermarkets); produce; meat (butcher shops); fishmongers; restaurants and similar establishments; open-air markets; fish market; and waterfront kiosks selling exclusively coconut water.
Stores predominantly selling processed foods	Retail sales of dairy products and cold cuts; bakery products; sweets and pastries shops with a focus on resale.
Stores predominantly selling ultra-processed foods	Mini-markets, grocery stores, and warehouses; snack bars, tea houses, and juice bars; bars and establishments specializing in serving beverages; convenience stores; candy shops (candy and sweets); waterfront kiosks, except for those selling exclusively coconut water; and district markets.

Source: Adapted from Silva et al. [22]. **Note:** Grouping of food retail stores was based on the Nova classification: Natural or minimally processed foods: foods obtained directly from plants or animals that undergo no changes after being harvested or caught, or that undergo only minimal processing (cleaning, grinding, drying, pasteurization) without the addition of salt, sugar, oils, or fats. Processed culinary ingredients: ingredients extracted from nature or from natural foods (oils, fats, salt, and sugar) used to season, to cook and to prepare recipes. Processed foods: products manufactured essentially with the addition of salt or sugar (or another substance of culinary use, such as oil or vinegar) to a natural or minimally processed food. Ultra-processed foods: industrial formulations made primarily from food-derived substances, often containing cosmetic additives (colors, flavorings, emulsifiers) to enhance palatability [28]. Methodological adjustment for kiosks: Given that the "kiosks" category is not included in the original Locais-Nova classification, an adjustment was made to include them. In general, kiosks were grouped under the "bars" category, as they share similar operational characteristics and product offerings, with a predominance of ultra-processed foods. An exception was made for kiosks selling exclusively coconut water, which were classified as a store predominantly selling fresh foods.

Table S2. Pearson's correlation between GeoSES Index and its dimensions with food retail stores types' availability in Joao Pessoa, Brazil, 2023–2024.

Variables	Fresh Foods (r)	Processed Foods (r)	Ultra-Processed Foods (r)
**GeoSES index**	0.85 (p-value<0.001)	-0.05 (p-value=0.88)	0.34 (p-value=0.27)
**Education**	0.83 (p-value<0.001)	-0.11 (p-value=0.73)	0.28 (p-value=0.37)
**Income**	0.85 (p-value<0.001)	-0.08 (p-value=0.80)	0.30 (p-value=0.34)
**Poverty**	-0.47 (p-value=0.12)	0.24 (p-value=0.45)	-0.07 (p-value=0.84)
**Material Deprivation**	0.85 (p-value<0.001)	0.06 (p-value=0.86)	0.41 (p-value=0.18)
**Mobility**	0.59 (p-value=0.04)	-0.27 (p-value=0.40)	0.12 (p-value=0.71)

Note: r=Pearson's correlation coefficient.
